# Modeling the Evolution of Biological Neural Networks Based on *Caenorhabditis elegans* Connectomes across Development

**DOI:** 10.3390/e25010051

**Published:** 2022-12-27

**Authors:** Hongfei Zhao, Zhiguo Shi, Zhefeng Gong, Shibo He

**Affiliations:** 1 College of Information Science and Electronic Engineering, Zhejiang University, Hangzhou 310027, China; 2 Key Laboratory of Collaborative Sensing and Autonomous Unmanned Systems of Zhejiang Province, Zhejiang University, Hangzhou 310058, China; 3 Department of Neurobiology, Zhejiang University School of Medicine, Hangzhou 310058, China; 4 Key Laboratory of Medical Neurobiology of the Ministry of Health of China, Key Laboratory of Neurobiology, Zhejiang University School of Medicine, Hangzhou 310058, China; 5 College of Control Science and Engineering, Zhejiang University, Hangzhou 310027, China

**Keywords:** biological neural networks, *C. elegans* connectomes, structural properties, network model, initial attractiveness, asymmetry

## Abstract

Knowledge of the structural properties of biological neural networks can help in understanding how particular responses and actions are generated. Recently, Witvliet et al. published the connectomes of eight isogenic *Caenorhabditis elegans* hermaphrodites at different postembryonic ages, from birth to adulthood. We analyzed the basic structural properties of these biological neural networks. From birth to adulthood, the asymmetry between in-degrees and out-degrees over the *C. elegans* neuronal network increased with age, in addition to an increase in the number of nodes and edges. The degree distributions were neither Poisson distributions nor pure power-law distributions. We have proposed a model of network evolution with different initial attractiveness for in-degrees and out-degrees of nodes and preferential attachment, which reproduces the asymmetry between in-degrees and out-degrees and similar degree distributions via the tuning of the initial attractiveness values. In this study, we present the well-preserved structural properties of *C. elegans* neuronal networks across development, and provide some insight into understanding the evolutionary processes of biological neural networks through a simple network model.

## 1. Introduction

Understanding wiring diagrams of the brain provides us with insights into how circuits respond to internal and external cues, and helps us understand how complex dynamics arise. In the past few decades, connectomes at synaptic resolution have been reconstructed for the nematode *C. elegans* [1,2,3,4], the central nervous system of a tadpole larva of *Ciona intestinalis* (L.) [5], the visual system, olfactory system, mushroom body (MB), and locomotion circuits of larval *Drosophila* [6,7,8,9], the optic medulla and the central brain of adult *Drosophila* [10,11,12,13] and the inner plexiform layer in the mouse retina [14]. Recently, Witvliet et al. fully reconstructed the connectomes of eight isogenic *C. elegans* hermaphrodites at different postembryonic ages, from hatching (birth) to adulthood, and investigated how the brain changes with age [4]. *C. elegans* develops from a fertilized egg through four larval stages (that is, four molt cycles) to become an adult. Under crowded conditions or in the absence of food, larvae can choose an alternative developmental pathway, becoming dauer larvae, which do not feed but can survive adverse conditions for several months. When living conditions improve and normal development is resumed, the animals exit the dauer larval stage and develop into the normal fourth larval stage before becoming adults. The developmental age of each animal was confirmed based on the described temporal cell division pattern exhibited by wild-type (N2) larva raised at 25 °C [4]. Obviously, there are stable and variable structures in chemical synaptic networks at different developmental ages.

Some studies have demonstrated that the *C. elegans* neuronal network has small-world properties [15,16,17] and network motifs [18,19]. Its tails of in-degree and out-degree distributions decay with an exponential, but not a power law [15,20]. However, it is unknown whether these structural and statistical properties are preserved from birth to adulthood, mainly due to the lack of connectomes at different developmental time points. The connectomes of *C. elegans* across development provide an opportunity for this study.

There are several network models available to study the mechanisms underlying the evolution of real networks (WWW, actor collaboration network, scientific citation networks, and social networks), such as the growth and preferential attachment mechanisms [21,22,23,24], initial attractiveness mechanism [23], the fitness mechanism [25,26], aging and cost mechanism [20,27], and the directional attachment and community structure model [28], to name a few. Berry and Temam proposed a network growth model in a three-dimensional space, which reproduced most of the structural properties exhibited by the *C. elegans* neuronal network, including its small-world structure [15]. In this model, the physical distance between two neurons was considered. However, they only used the adult *C. elegans* neuronal network, including the connections formed by gap junctions and chemical synapses. Fortunately, more connectomes have been reconstructed, providing important insights in to the design principles of biological neural networks [3,4,5,6,7,8,9,10,12,14]. Furthermore, several researchers have made use of the structural features found in biological neural networks to design deep-learning architectures and algorithms for performing approximate-similarity (or nearest-neighbor) searches in machine learning [29], causal inference for motor estimation [30], natural language processing (NLP) tasks [31], or enabling auditable autonomy for an autonomous vehicle control system [32]. Therefore, it is meaningful to study the structural properties of biological neural networks and design a proper network model that can reproduce these properties.

In this study, we analyzed the basic structural properties of the biological neural networks of *C. elegans* at different developmental ages. Structural properties such as small-world properties, asymmetry between in-degrees and out-degrees of nodes, and degree distributions following neither a Poisson distribution nor a pure power-law distribution, were found to be well-preserved during development. On the basis of the asymmetry between in-degrees and out-degrees, we proposed a model of network evolution with different initial attractiveness for the in-degrees and out-degrees of nodes. According to the degree distribution of the Barabási–Albert (BA) model without the growth mechanism [21], we introduced the mechanism of preferential attachment into our model. In other words, the probability that a node gets a new edge depends on the initial attractiveness and its in-degree or out-degree. Finally, a network with asymmetry between in-degrees and out-degrees, as generated by this model, had an average shortest path length and a degree distribution shape that were similar to those of the biological neural networks of *C. elegans*. This was achieved through tuning the initial attractiveness values for in-degrees and out-degrees of nodes. In this study, we present the well-preserved structural properties of *C. elegans* neuronal networks during development, and provide some insights into understanding the evolutionary processes of biological neural networks through the use of a simple network model.

## 2. Materials and Methods

### 2.1. Biological Neural Networks of *C. elegans* throughout Development

Witvliet et al. [4] used serial-section electron microscopy to reconstruct the brains of eight isogenic *C. elegans* hermaphrodites, specifically, their circumpharyngeal nerve ring and ventral ganglion, at different postembryonic ages from birth to adulthood. *C. elegans* proceeds through four molt cycles before becoming an adult. The first larval stage (L1) is from hatching (birth) to the first molt; the second larval stage (L2) is from the first molt to the second molt; the third larval stage (L3) is from the second molt to the third molt; and the fourth larval stage (L4) is from the third molt to the fourth molt. After experiencing the fourth molt, *C. elegans* becomes a mature adult.

**Figure 1:**
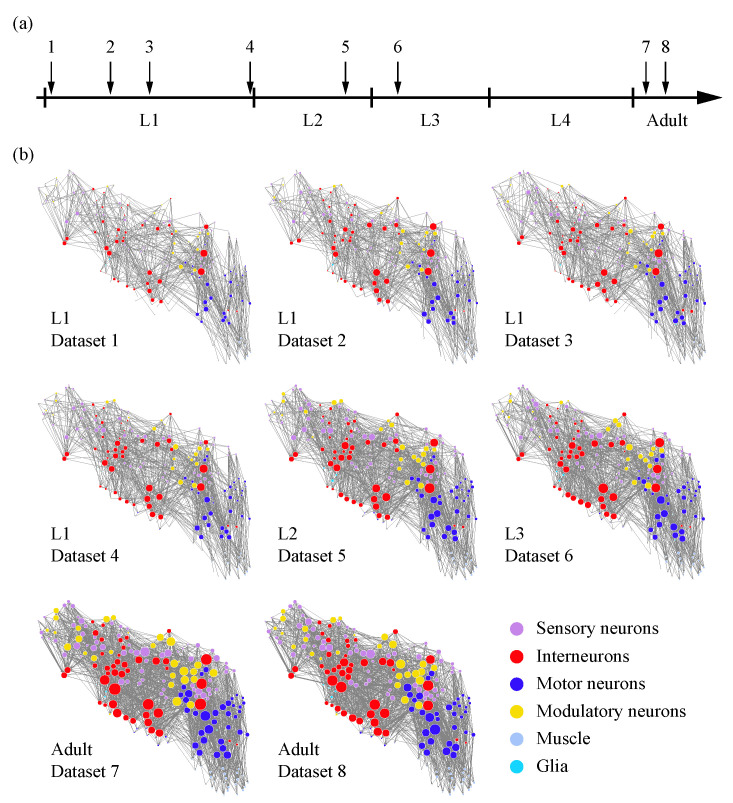
Visualization of the biological neural networks of *C. elegans* throughout development. (**a**) Developmental timeline of the eight reconstructed *C. elegans* hermaphrodites. (**b**) Wiring diagrams for the eight individuals. Each circle represents a cell. Circle color denotes cell type. Circle size is proportional to the connection number of a cell. Each line represents a connection with at least one chemical synapse between two cells, and the line width is proportional to the synapse number per connection. L1: the first larval stage; L2: the second larval stage; L3: the third larval stage; L4: the fourth larval stage.

**Figure A1:**
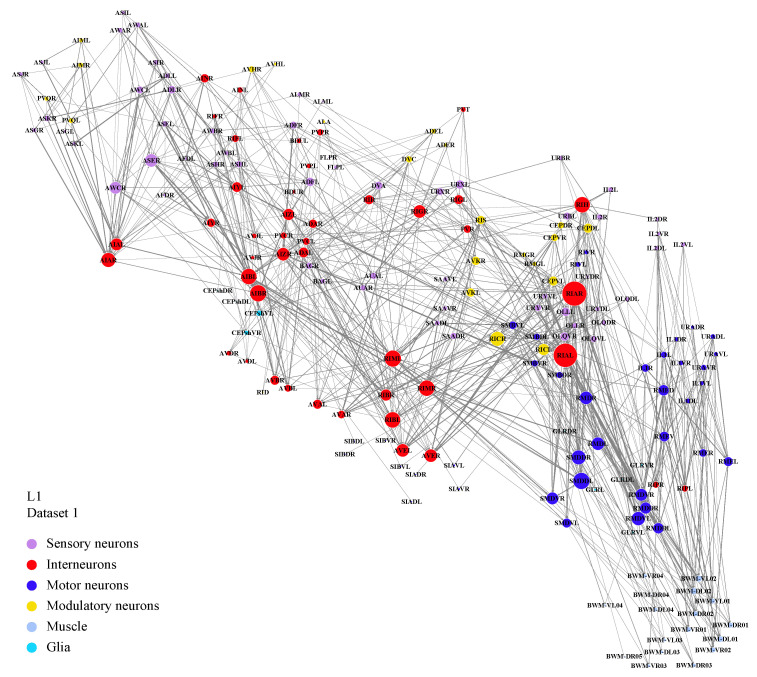
Closeup of a larval brain connectome at L1 stages (dataset 1). Each circle represents a cell. Circle colour denotes cell type. Circle size is proportional to the connection number of a cell. Each line represents a connection with at least one chemical synapse between two cells, and line width is proportional to synapse number per connection.

**Figure A2:**
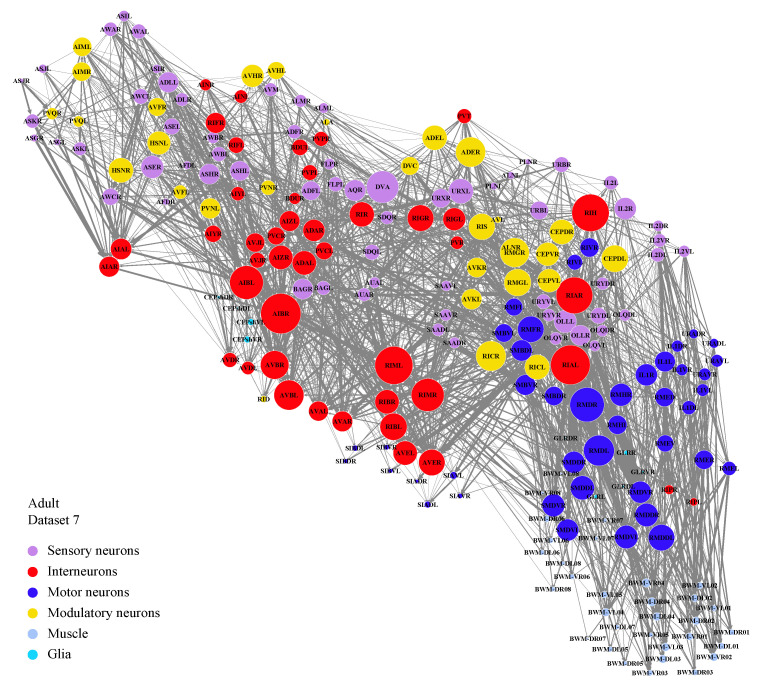
Closeup of an adult brain connectome (dataset 7). Each circle represents a cell. Circle colour denotes cell type. Circle size is proportional to the connection number of a cell. Each line represents a connection with at least one chemical synapse between two cells, and line width is proportional to synapse number per connection.

The developmental ages of the eight samples are listed in Table 1. Connectivity matrices for the eight datasets are available at https://www.nemanode.org/ or https://www.nature.com/articles/s41586-021-03778-8#Sec37, and the former includes connections formed by electrical synapses (gap junctions). A connection or edge is defined as a pair of cells connected by one or more chemical synapses. Electrical synapses were partially annotated and incomplete; thus, we used chemical synaptic networks, in which connections were formed by chemical synapses between neurons, muscles, and glia. Chemical synapses exhibit clear directionality, and self-loops are rare in these datasets, so we treated these chemical synaptic networks as directed networks without self-loops. Visualizations of these biological neural networks of *C. elegans* are shown in Figure 1b and Appendix A Figure A1 and Figure A2, in which the cells’ coordinates are roughly the same, with overlapping cells manually separated.

### 2.2. Network Structure Analysis

The common structural characteristics of a network, aside from the number of nodes, the number of edges, and the network density, include the average degree, average shortest path length, average clustering coefficient, and degree distribution. The average shortest path length and average clustering coefficient are two key features used to determine whether a network has small-world properties. A network has small-world properties if it has a comparable average shortest path length and a higher average clustering coefficient with regard to a random network. The in-degree $k_{i}^{in}$ or out-degree $k_{i}^{out}$ of the node *i* is the number of edges pointing to or pointing out of node *i*. The degree $k_{i}$ of node *i* is the sum of $k_{i}^{in}$ and $k_{i}^{out}$. The average in-degree $<k^{in}>$ or average out-degree $<k^{out}>$ is the average of the $k_{i}^{in}s$ or $k_{i}^{out}s$ over all nodes in the network. For a directed network, the average degree is the average in-degree or out-degree. p(k) is the probability density function of $k^{in}$ or $k^{out}$. Furthermore, the asymmetry index between the in-degrees and out-degrees of a node is an indicator used to express the tendency of the node to have unbalanced out-degrees and in-degrees. Our calculation of the asymmetry index was modified from that presented in [15]. The following is the formula used for the calculation of these parameters.

The density ρ of a directed network is calculated by

(1) $$\rho =\frac{M}{N(N-1)},$$

where *N* is the number of nodes and *M* is the number of edges in the network.

The average in-degree $<k^{in}>$ or out-degree $<k^{out}>$ of the network is equal to the number of edges of the network *M* divided by the number of nodes *N*, which is

(2) $$<k^{in}>=<k^{out}>=\frac{M}{N}.$$

The average shortest path length *L* is

(3) $$L=\frac{1}{N(N-1)}\sum_{i,j,i\neq j}d_{ij},$$

where $d_{ij}$ is the shortest path length between node *i* and *j*.

The clustering coefficient $C_{i}$ of a node *i* with $k_{i}$ neighbors is defined by

(4) $$C_{i}=\frac{2E_{i}}{k_{i}\left(k_{i}-1\right)},$$

where $E_{i}$ is the number of edges among the $k_{i}$ neighbors of node *i*, excluding the edges between the neighbors and node *i* itself. The average clustering coefficient <*C*> is

(5) $$<C>=\frac{1}{N}\sum_{i}C_{i}.$$

We used the quantitative metric of “small-world-ness” defined by [33]. Let $C_{rand}$ and $L_{rand}$ be the corresponding values obtained by averaging over a set of randomized networks. *S* is the ratio of $C/C_{rand}$ to $L/L_{rand}$, which is

(6) $$S=\frac{<C>L_{rand}}{C_{rand}L}.$$

The criterion for “small worldness” is S>1, indicating that the network has a higher average clustering coefficient when compared to randomized networks, while still maintaining the average shortest path length, as found in random networks.

For the degree distribution, the probability density function p(k) is equal to the number of nodes with *k* divided by the number of total nodes *N*, which is

(7) $$p\left(k\right)=\frac{N(k)}{N},$$

where N(k) is the number of nodes with *k*, and *k* refers to either the in-degree $k^{in}$ or the out-degree $k^{out}$ in our study. The cumulative distribution function P(k) is defined by

(8) $$P\left(k\right)=P\left(x\geq k\right)=\sum_{x\geq k}^{}p\left(x\right).$$

The asymmetry index $\alpha_{i}$ of node *i* is defined by

(9) $$\alpha_{i}=\frac{k_{i}^{in}-k_{i}^{out}}{k_{i}^{in}+k_{i}^{out}}.$$

The value of $\alpha_{i}$ is greater than 0 when the node *i* has a larger in-degree value than the out-degree value. The $\alpha_{i}$ is less than 0 when the node *i* has a smaller in-degree value than the out-degree value. The $\alpha_{i}$ is equal to 0 when the node *i* has the same in-degree value as the out-degree value. The average value of the $\alpha_{i}s$ throughout the network, <α>, is

(10) $$<\alpha >=\frac{1}{n}\sum_{i}\alpha_{i}.$$

### 2.3. Generation of Randomized Networks

In the analysis of small-world properties, the values of real networks need to be compared with those of randomized networks. We used two random network models. The Erdös–Rényi random model generates randomized networks with the same number of nodes and edges as a real network through randomly choosing *M* edges from [N(N−1)]/2 (undirected) or N(N−1) (directed) possible edges, where *N* is the number of nodes and *M* is the number of edges in the real network. The degree-preserving randomization (DPR) model keeps the degree of nodes in the network unchanged, that is, it generates randomized networks with the given degree sequence (also known as the first-order null model). Starting with a real network, we randomly chose two existing edges (u1, v1) and (u2, v2) every time (u1, v1, u2, and v2 are nodes), and deleted the two edges (u1, v1) and (u2, v2), followed by adding two new edges, (u1, v2) and (u2, v1). Switching was prohibited if either of the edges (u1, v2) or (u2, v1) already existed or the node u1 was v2 or v1 was u2. This process was repeated until the network was well randomized. Through the use of this rewiring algorithm, the generated randomized networks had the same degree sequence, without parallel edges or self-loops.

### 2.4. Data Analysis or Statistical Analysis

Visualizations of the biological neural networks of *C. elegans* throughout development were generated using Gephi 0.9 software. We used the NetworkX package in python 3.7.6 to analyze the structural properties of networks, such as their small-world properties, degree distribution, and numerical simulations.

## 3. Results

### 3.1. Small-World Properties, Asymmetry between In-Degrees and Out-Degrees of C. Elegans Neural Networks throughout Development

The structural properties of the adult *C. elegans* neuronal network have been studied previously, such as small-network properties [16,17,34], the asymmetry between in-degrees and out-degrees [15], self-similar and fractal properties [17], and higher-order clustering [35]. Witvliet et al. [4] reconstructed the brains of eight isogenic *C. elegans* individuals across postnatal stages, and observed non-uniform synapse addition, stereotyped and variable connections, stable interneuron connections, increase in feedforward signaling and discernible modularity during maturation. Moreover, there may be other statistical and structural changes with age.

**Figure 2:**
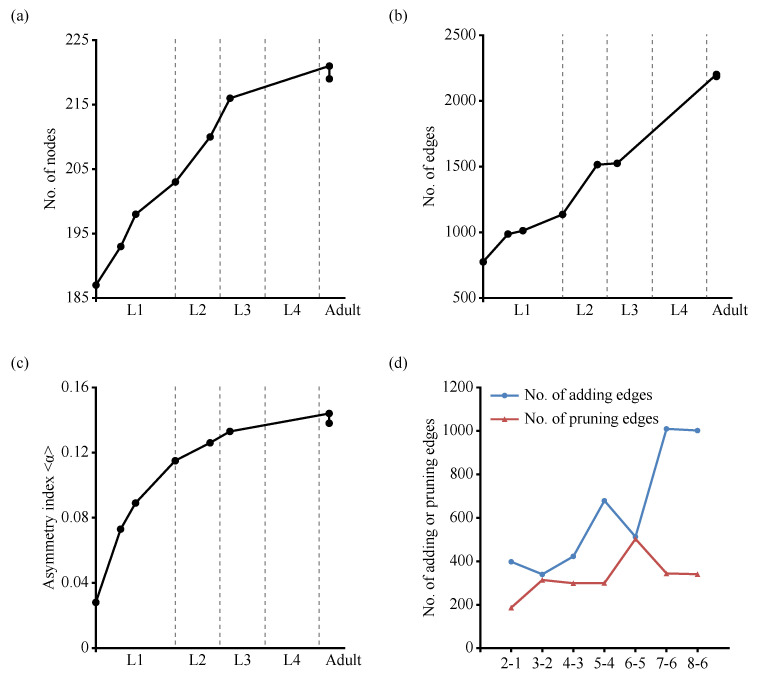
In the developing *C. elegans* brain, the number of edges and asymmetry between in-degrees and out-degrees increased with age. (**a**) The number of nodes (cells) in each dataset. (**b**) The number of edges (connections) in each dataset. (**c**) Index reflecting the asymmetry between in-degrees and out-degrees in each dataset. (**d**) The number of adding or pruning edges from an earlier connectome to the subsequent one. An edge was considered to be added when it was absent from the earlier connectome (such as dataset 6) and present in the subsequent one (such as in dataset 7 and dataset 8); an edge was considered to be pruned when it was present in the earlier connectome (such as dataset 6) and absent from the subsequent one (such as in dataset 7 and dataset 8).

Some studies have indicated that the adult *C. elegans* neuronal network has small-world properties [16,17,34]. However, it is not clear whether these biological neural networks of *C. elegans* throughout development have small-world attributes. Thus, we first calculated the average shortest path length *L* and the average clustering coefficient <*C*>, referring to Equations (3)–(5). We used the quantitative metric *S* of “small worldness” defined by [33]. As shown in Table 2, compared to the values obtained by averaging over 1000 randomized networks with the same number of nodes *N* and the number of edges *M*, as generated by the Erdös–Rényi random model or the degree-preserving randomization (DPR) model, these biological neural networks of *C. elegans* had a much higher <*C*> while maintaining a comparable *L* and S>1, indicating that these *C. elegans* neuronal networks across development possessed small-world properties. Furthermore, from birth to adulthood, there were many more new edges generated due to new synapse formation than new nodes generated via neurogenesis (an approximately 17% increase in nodes, and an approximately 182% increase in edges; see Figure 2a,b). The average clustering coefficient <*C*> slightly increased over time. The asymmetry between in-degrees and out-degrees over the *C. elegans* neuronal network increased with age (Table 2 and Figure 2c), suggesting that the neuronal networks of *C. elegans* increased their ability to integrate information during development.

**Figure 3:**
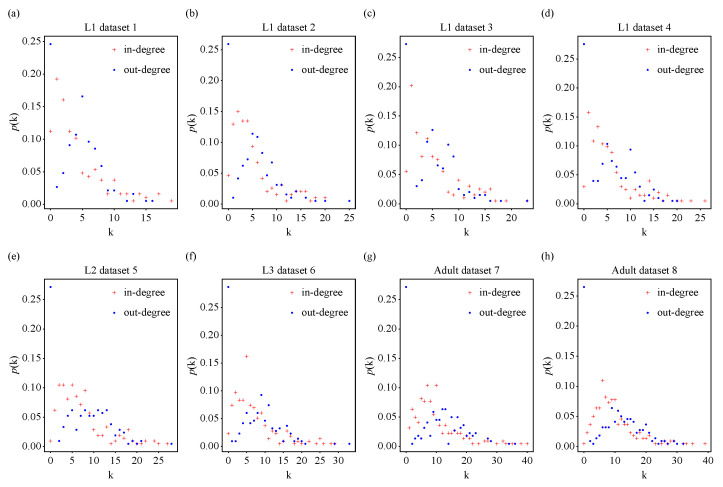
Linear–linear plot of the probability density function p(k) of in-degrees and out-degrees for biological neural networks of *C. elegans* throughout development. Red plus signs represent in-degree data and blue dots represent out-degree data.

**Figure A3:**
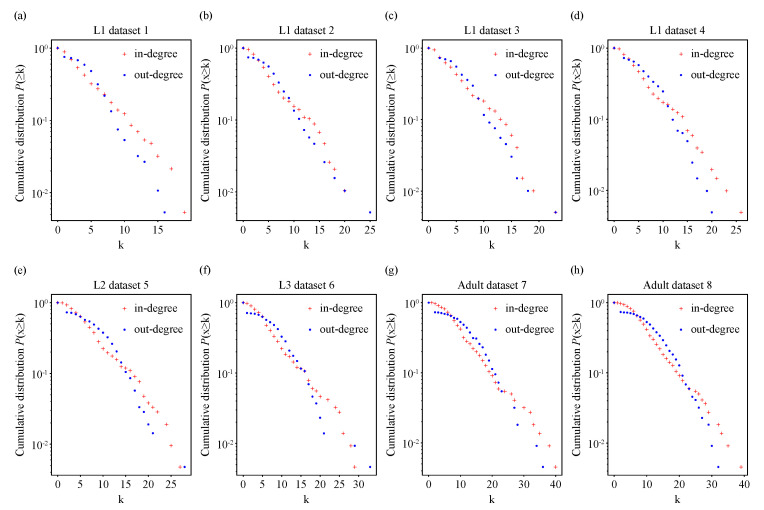
Linear–log plot of the cumulative distribution P(x≥k) of in-degrees or out-degrees for biological neural networks of *C. elegans* across development. If the distribution would have a exponential tail, then it would fall on a straight line in a linear–log plot. Red plus signs represent in-degree data and blue dots represent out-degree data.

**Figure A4:**
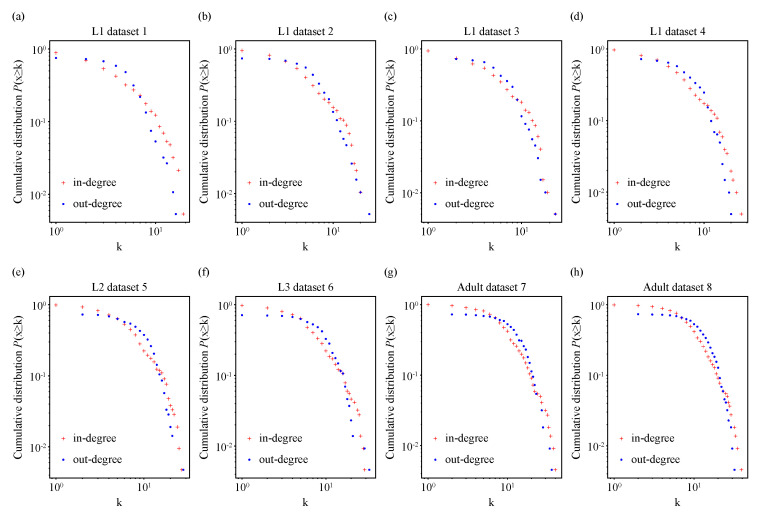
Log–log plots of the cumulative distribution P(x≥k) of in-degrees or out-degrees for biological neural networks of *C. elegans* across development. If the distribution would have a power-law tail, then it would fall on a straight line in a log–log plot. Red plus signs represent in-degree data and blue dots represent out-degree data.

We also analyzed the statistical property of in-degrees and out-degrees for these biological neural networks of *C. elegans* during development, namely, the degree distribution. Linear–linear plots of the probability density function of in-degrees and out-degrees, as well as linear–log plots and log–log plots of the cumulative distribution of in-degrees and out-degrees for biological neural networks of *C. elegans* throughout development are displayed in Figure 3, Figure A3 and Figure A4, respectively. Unlike Poisson distributions or power-law distributions, there was a left-skewed peak around the average in-degree <$k^{in}$> or out-degree <$k^{out}$> in the linear–linear plots of the probability density function of in-degrees and out-degrees (Figure 3), and the maximum in-degree or out-degree value of each developmental stage increased with age. Compared with the pure power-law distribution, which falls on a straight line in a log–log plot, the distributions could be better approximated by exponential decay (Figure A3 and Figure A4), which is in agreement with the results obtained in the adult *C. elegans* neuronal network [20].

From birth to adulthood, in addition to an increase in the number of nodes and edges, synapse pruning is a hallmark of early development in mammals [4,36]. An edge is deemed to be added when it is absent from the earlier connectome (such as in dataset 1) and present in the subsequent one (such as dataset 2). An edge is deemed to be pruned or removed when it is present in the earlier connectome (such as in dataset 6) and absent from the subsequent one (such as dataset 7 or 8). Comparing dataset 7 and dataset 8, both of which were adult connectomes, they had 1469 common edges, whereas dataset 7 had 722 edges that were not shared by dataset 8, and dataset 8 had 717 edges that were not shared by dataset 7. This suggests connectivity differences between individuals. From the earlier age to the subsequent one (such as 2-1 and 5-4 in Figure 2d), old edges (present in the earlier dataset) were removed, as new edges were added. Furthermore, edges with five synapses were absent from stage L3 to adulthood. Therefore, some edges were removed, despite the connectivity differences between individuals and rare pruning in the *C. elegans* brain.

### 3.2. A Model of Network Evolution with Different Initial Attractiveness for In-Degrees or Out-Degrees

The biological neural networks of *C. elegans* across development revealed some essential characteristics that underlie brain evolution, such as, the addition of more edges compared to the change in the node number, the asymmetry between in-degrees and out-degrees, as well as the fact that the degree distribution did not exhibit a Poisson distribution or a pure power-law distribution. Moreover, the *C. elegans* neuronal networks at each developmental time point exhibited a comparable number of nodes and edges. Based on these characteristics, we proposed a network evolution model with different initial attractiveness for the in-degrees and out-degrees of nodes, which could be used to generate a random network with *N* nodes and *M* edges. The different initial attractiveness for in-degrees and out-degrees contributes to the asymmetry between in-degrees and out-degrees of nodes.

**Figure 4:**
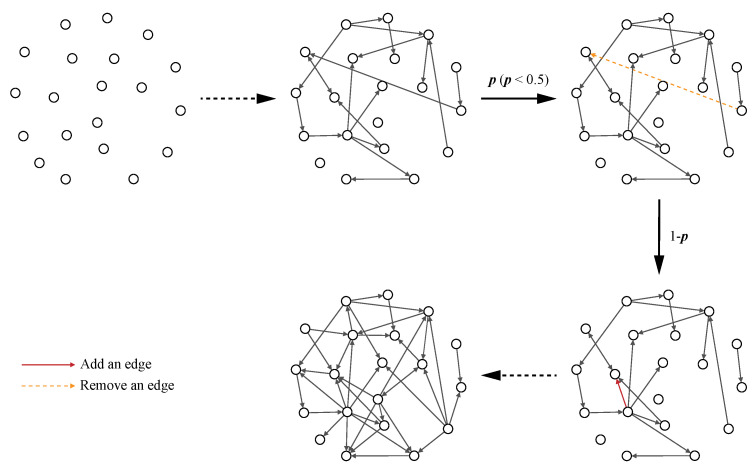
Schematic illustration of the model for N=20, M=40, a=2, b=3, and p=0.1. Starting with *N* isolated nodes, at each time step, we perform one of the following operations. With a probability of 1−p, a new directed edge is added to the the network using preferential attachment. With probability *p* (p<0.5), we randomly select an edge and remove it, if the network has edges. Self-loops and duplicate edges are forbidden.

In the model, we start with *N* isolated nodes, of which the initial attractiveness is *a* for in-degrees and *b* for out-degrees, and at each time step we perform one of the following two steps (see Figure 4).

(1) With a probability of p(p<0.5), we randomly remove an edge from the network. For this, there must be at least one edge in the network.

(2) With a probability of 1−p, we add a new edge. The probability that a node *i* will increase its in-degree (or out-degree) depends on $k_{i}^{in}$ (or $k_{i}^{out}$) and the initial attractiveness, *a* (or *b*). The new edge $e_{ij}$ is added from node *i* to node *j*, and nodes *i* and *j* are selected with a probability of

(11) $$\prod_{i^{out}}=\frac{k_{i}^{out}+b}{\sum_{x}k_{x}^{out}+b},$$

and

(12) $$\prod_{j^{in}}=\frac{k_{i}^{in}+a}{\sum_{x}k_{x}^{in}+a},$$

respectively, where *a* and *b* are the initial attractiveness for the in-degrees and out-degrees of nodes in the network, respectively.

According to the continuum theory mentioned in [22], we take the change in the in-degree $k_{i}^{in}$ of node *i* as an example, and the probability $\prod_{k_{i}^{in}}^{}$ can be interpreted as the rate at which $k_{i}^{in}$ increases. Therefore, the change in $k_{i}^{in}$ consists of the following two steps.

(1) The removal of an edge with probability *p*:

(13) $${\frac{\partial k_{i}^{in}}{\partial t}}_{\left(1\right)}=-p\frac{1}{E},$$

where *E* is the number of edges in the network at time *t*.

(2) The addition of a new edge with probability 1−p:

(14) $${\frac{\partial k_{i}^{in}}{\partial t}}_{\left(2\right)}=\left(1-p\right)\frac{k_{i}^{in}+a}{\sum_{j}k_{j}^{in}+a}.$$

By adding the contributions of the two processes, we obtain

(15) $$\frac{\partial k_{i}^{in}}{\partial t}=\left(1-p\right)\frac{k_{i}^{in}+a}{\sum_{j}k_{j}^{in}+a}-p\frac{1}{E}.$$

In the rate equation above, the number of edges *E* varies with time *t* as E(t)=(1−2p)t, and $\sum_{j}k_{j}^{in}+a=\left(1-2p\right)t+Na$. Using as an initial condition, the in-degree of a node *i* added at time $t_{i}$, $k_{i}^{in}\left(t_{i}\right)=1$, the solution of the equation above for $k_{i}^{in}\left(t\right)$ has the form

(16) $$k_{i}^{in}\left(t\right)=-a+C_{0}{\left(A+Bt\right)}^{\frac{1}{B}}-{\left(A+Bt\right)}^{\frac{1}{B}}\int \frac{p}{(1-2p)t}{\left(A+Bt\right)}^{\frac{1}{B}}dt,$$

where $A=\frac{Na}{(1-2p)t}$ and $B=\frac{1-2p}{1-p}$, $C_{0}$ is a constant related to $k_{i}^{in}\left(t_{i}\right)=1$.

Given a special case where *p* is small enough to reach zero, the third term on the left side of Equation (16) is negligible; thus

(17) $$k_{i}^{in}\left(t\right)=\left(1+a\right)\frac{Na+t}{Na+t_{i}}-a\;\left(t\geq t_{i}\right).$$

In this case, when *t* is large, the in-degree of each node increases linearly with time *t*.

The above procedure can also be applied to analyze the change in node out-degrees over time, except for the case in which the initial attractiveness for out-degrees is *b*.

### 3.3. Simulation

#### 3.3.1. The Asymmetry between In-Degrees and Out-Degrees of Nodes in a Network Is Closely Related to the Initial Attractiveness of In-Degrees and Out-Degrees

The networks generated by the above model with different initial attractiveness values for in-degrees and out-degrees exhibit asymmetry between in-degrees and out-degrees. If the initial attractiveness of the in-degrees, *a*, is larger than that of the out-degrees, *b*, the asymmetry index <α> will be greater than zero, and vice versa. <α> will be close to zero if *a* is equal to *b*.

**Figure 5:**
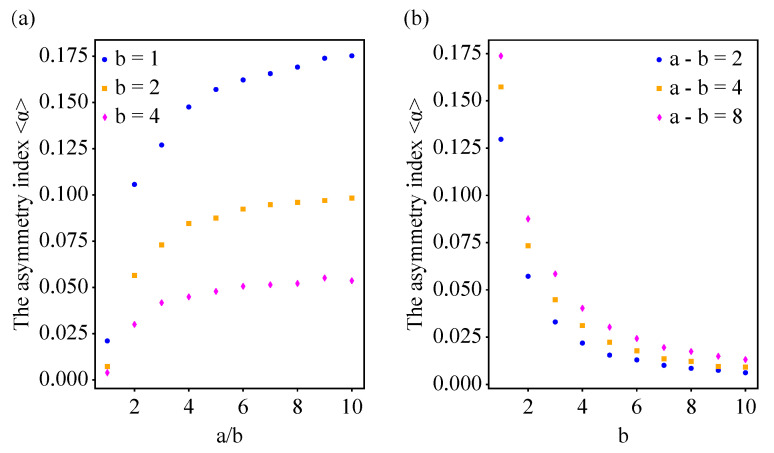
Numerical simulations investigating the impact of the initial attractiveness of in-degrees and out-degrees of nodes on the asymmetry between in-degrees and out-degrees of a network. (**a**) The asymmetry index increases with the ratio of the initial attractiveness *a* of in-degrees to the initial attractiveness *b* of out-degrees. The symbols correspond to b=1 (circles), b=2 (squares), b=4 (diamonds). We used N=300, M=3000 and p=0.1. The asymmetry index decreases with the increase in *b* for out-degrees when the ratio is fixed. (**b**) The asymmetry index <α> decreases with *b* for out-degrees, when the difference between *a* and *b* is fixed. The symbols correspond to a−b=2 (circles), a−b=4 (squares), a−b=8 (diamonds). We used N=300, M=3000 and p=0.1. The greater the difference between *a* and *b*, the greater the asymmetry index <α>.

We investigated the impact of *a* and *b* on the asymmetry between in-degrees and out-degrees of nodes in a network (Figure 5). We found that the asymmetry index <α> increased with the ratio of *a* to *b* (Figure 5a). When the ratio of *a* and *b* was fixed, the asymmetry index <α> dramatically decreased with the increase in *b*. Similarly, when the difference between *a* and *b* was fixed, the asymmetry index <α> decreased with the increase in *b* (Figure 5b). Note, however, that for *b* values greater than 1, or even a/b=10, the asymmetry index was less than 0.100. Therefore, a proper asymmetry index for a modeling network is obtained by tuning the ratio or the difference of the initial attractiveness *a* for in-degrees and *b* for out-degrees.

#### 3.3.2. The Shape of the Degree Distribution Changes with the Initial Attractiveness of In-Degrees and Out-Degrees

**Figure 6:**
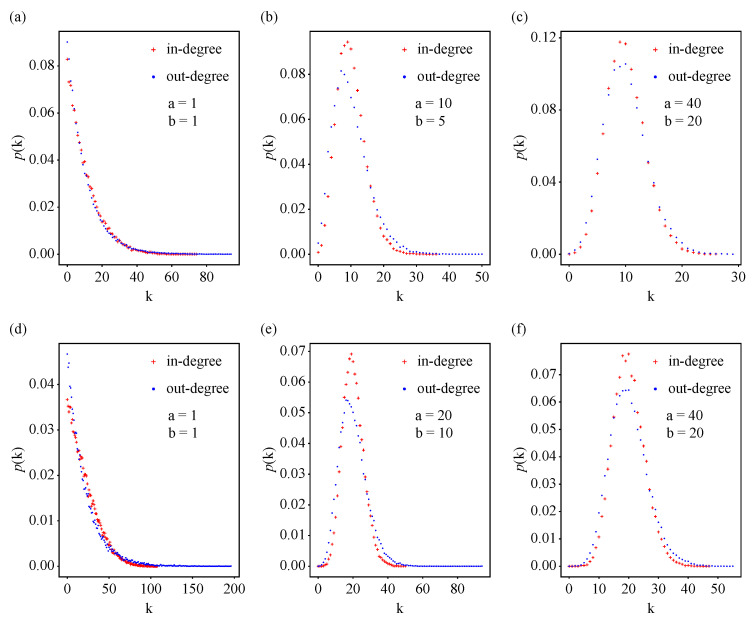
Numerical simulations investigating the impact of the initial attractiveness of the in-degrees and out-degrees of nodes on the degree distribution of a network. (**a**–**c**) The probability density function p(k) of networks with N=300, M=3000, and p=0.1. The initial attractiveness *a* for in-degrees and *b* for out-degrees were a=b=1, a=10,b=5 and a=40,b=20, respectively. (**d**–**f**) The probability density function p(k) of networks with N=300, M=6000, and p=0.1. The initial attractiveness *a* for in-degrees and *b* for out-degrees were a=b=1, a=20,b=10 and a=40,b=20, respectively. The initial attractiveness for in-degrees and out-degrees can vary the shape of the degree distribution. Results for the model were averaged over 100 realizations.

In model B of the scale-free model [21], which keeps preferential attachment and eliminates the growth mechanism, the shape of the degree distribution changes with the number of edges when the number of nodes is fixed. After a transient period, the node degrees converge to the average degree, and the degree develops a peak. Furthermore, in the extended BA model [22], the degree distribution develops an exponential tail as the rewiring probability q→1. Our proposed model incorporates preferential attachment and the initial attractiveness of in-degrees and out-degrees, enabling us to generate a directed network with *N* nodes and *M* edges. Here, we investigated how the shape of the degree distribution varied by performing numerical simulations. We observed that the initial attractiveness for in-degrees and out-degrees could lead to changes in the shape of degree distribution. We identified three cases: (1) When the initial attractiveness of in-degrees and out-degrees is equal to 1, that is, a=b=1, p(k) decays exponentially (Figure 6a,d). (2) When the initial attractiveness of in-degrees and out-degrees is greater than 1 and less than the average degree, p(k) has a peak at the left of the average degree (Figure 6b,e). (3) When the initial attractiveness of in-degrees and out-degrees is equal to or greater than the average degree, p(k) has a peak at the average degree (Figure 6c,f). Moreover, the initial attractiveness also affects the maximum in-degree or out-degree of nodes, which is explained by Equations (11) and (12). Accordingly, for a network with *N* nodes and *M* edges, we can obtain various shapes for the degree distribution by tuning the initial attractiveness values.

**Figure 7:**
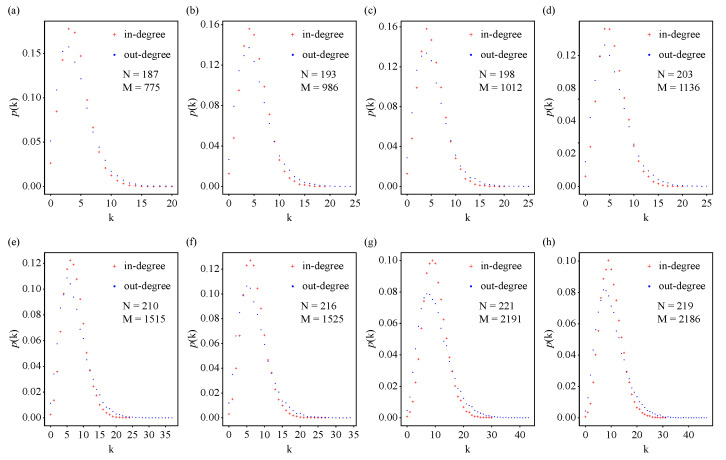
The probability density function p(k) of the degree distribution for networks generated using our model matching *C. elegans* neuronal networks. Based on the maximum in-degree and out-degree and the location of the peak in the *C. elegans* neuronal networks, the initial attractiveness values for in-degrees and out-degrees were a=12 and b=5, respectively. The results for the model were averaged over 100 realizations.

The degree distributions of these *C. elegans* biological neural networks exhibited a peak around the average degree and an exponential tail. Therefore, we plotted the degree distribution of networks generated by our model for the corresponding *C. elegans* neuronal networks (Figure 7). Based on the maximum in-degree and out-degree and the location of the peak, *a* and *b* were selected as a=12 and b=5, respectively. We reproduced a similar-shaped degree distribution for the corresponding *C. elegans* neuronal network. Nevertheless, there was a large difference when the out-degree was zero. This was due to the fact that most sensory neurons in the *C. elegans* neuronal networks only have out-degrees, which is closely related to the function of neurons.

## 4. Discussion

Wiring diagrams of the brain provide a structural basis for understanding their function and dynamics, but little is known about the common structural properties of biological neural networks during development. In this study, we first analyzed the structural properties of the biological neural networks of *C. elegans* across development to investigate the changes in these structures with age. Second, based on the structural properties of these *C. elegans* neuronal networks, we proposed a simple network model to reproduce properties of these biological neural networks. Third, we applied the proposed model to simulate the impact of initial attractiveness on the asymmetry between the in-degrees and out-degrees of nodes and the degree distribution. Our findings indicated that biological neural networks preserve several structural properties during development, and provided some insight into understanding the evolutionary processes of biological neural networks through the use of a simple network model.

From birth to adulthood, some changes occurred in the biological neural networks of *C. elegans*, including increase in the number of neurons and connections, stereotyped and variable connections. However, they retained some structural properties that played an important role in circuit dynamics and brain function. For example, the small-world properties indicated the efficient processing of information in the brain. The asymmetry between in-degrees and out-degrees of nodes showed that various types of cells (neurons, glia, or muscle cells) in the brain had different connections and functions. We speculate that such well-preserved structural properties found in *C. elegans* neuronal networks throughout development are conserved in other biological neural networks, and may be applied in the design of architectures and algorithms in artificial neural networks.

A statistical characteristic of a network is the probability density function p(k) of the degree distribution, which represents the fraction of the number of nodes that have *k* edges in the network. We showed that the degree distribution of *C. elegans* neuronal networks throughout development did not follow a Poisson distribution or a purely power-law distribution. In these biological neural networks, there was a left-skewed peak around the average in-degree or out-degree of the degree distribution, and the cumulative distribution decayed exponentially. In the extended BA model [22], as q→1, where the rewiring process is dominant, p(k) develops an exponential tail. For this reason, we proposed a directed network model with initial attractiveness and preferential attachment, in order to generate a network with *N* nodes and *M* edges. By tuning the initial attractiveness values of the in-degrees and out-degrees of nodes, we reproduced the asymmetry and a similar degree distribution to that of the *C. elegans* neuronal network. However, the average clustering coefficient of the network generated by this model was much smaller than that of the real network, and the asymmetry index was relatively small, although it was larger than zero. This is because our proposed model did not take other constraints into account. For example, the initial attractiveness values of different types of cells (sensory neurons, interneurons, motor neurons, glia, and muscle cells) were different. Neurons that execute the same function tend to have similar connections, referring to the duplication-divergence model of protein interaction networks [37]. We did not classify the types and functions of nodes in our model. We could reproduce more similar structural properties of biological neural networks if more constraints on the model were considered. The proposed model provides the basis for understanding the evolution of biological neural networks during maturation.

Further research will improve this model by taking more constraints into account, and investigate the application of these modeled neural networks to deep learning or machine learning.

## 5. Conclusions

During maturation, the biological neural network of *C. elegans* retains some structural properties that are essential for brain dynamics and functions. We proposed a network model that reproduced some structural properties of biological neural networks, such as the asymmetry between in-degrees and out-degrees of nodes and the degree distribution, which may provide some insight into understanding the evolutionary processes of biological neural networks.

## Figures and Tables

**Table 1:** The developmental ages of the eight samples. L1: the first larval stage; L2: the second larval stage; L3: the third larval stage; L4: the fourth larval stage.

Stages	L1				L2	L3	Adult	
**Dataset**	**1**	**2**	**3**	**4**	**5**	**6**	**7**	**8**
**Developmental age**	0	5	8	16	23	27	50	50

**Table 2:** Structural properties of biological neural networks of *C. elegans* throughout development. *C*: average clustering coefficient, the average probability that two nodes that are connected to the same third node are also connected to each other; *L*: average shortest path length, the average minimum number of edges separating any two nodes; $<C{>}^{ER}$, $L^{ER}$, $<C{>}^{DPR}$, $L^{DPR}$: the corresponding values obtained by averaging over 1000 randomized networks generated by the Erdös–Rényi (ER) model or degree-preserving randomization (DPR) model; $S^{ER}$, $S^{DPR}$: the ratio of $<C>/<C{>}^{ER}$ to $L/L^{ER}$ or the ratio of $<C>/<C{>}^{DPR}$ to $L/L^{DPR}$. The quantitative criteria for “small worldness” is $S^{ER}>1$ [33].

Stages	L1				L2	L3	Adult	
**Dataset**	**1**	**2**	**3**	**4**	**5**	**6**	**7**	**8**
*N*	187	193	198	203	210	216	221	219
*M*	775	986	1012	1136	1515	1525	2191	2186
ρ	0.022	0.027	0.026	0.028	0.035	0.033	0.045	0.046
<k>	4.144	5.109	5.111	5.596	7.214	7.060	9.914	9.982
<C>	0.109	0.125	0.122	0.120	0.154	0.153	0.184	0.190
*L*	2.536	2.356	2.387	2.365	2.418	2.201	2.069	2.058
$<C{>}^{ER}$	0.022	0.027	0.026	0.028	0.035	0.033	0.045	0.046
$L^{ER}$	3.669	3.347	3.363	3.242	2.910	2.952	2.598	2.588
$<C{>}^{DPR}$	0.038	0.047	0.045	0.047	0.057	0.058	0.075	0.074
$L^{DPR}$	2.249	2.265	2.188	2.203	2.043	1.988	1.855	1.840
$S^{ER}$	7.17	6.58	6.61	5.87	5.30	6.22	5.13	5.19
$S^{DPR}$	2.54	2.56	2.49	2.38	2.28	2.38	2.20	2.30
<α>	0.028	0.073	0.089	0.115	0.126	0.133	0.144	0.138
